# Mitophagy and Mitochondria Biogenesis Are Differentially Induced in Rat Skeletal Muscles during Immobilization and/or Remobilization

**DOI:** 10.3390/ijms21103691

**Published:** 2020-05-23

**Authors:** Christiane Deval, Julie Calonne, Cécile Coudy-Gandilhon, Emilie Vazeille, Daniel Bechet, Cécile Polge, Daniel Taillandier, Didier Attaix, Lydie Combaret

**Affiliations:** 1 Université Clermont Auvergne, INRAE, UNH, Unité de Nutrition Humaine, CRNH Auvergne, 63000 Clermont-Ferrand, France; christiane.deval@inrae.fr (C.D.); Cecile.Coudy-Gandilhon@inrae.fr (C.C.-G.); daniel.bechet@inrae.fr (D.B.); cecile.polge@inrae.fr (C.P.); daniel.taillandier@inrae.fr (D.T.); didier.attaix@inrae.fr (D.A.); 2 Department of Medicine, Université de Fribourg, CH-1700 Fribourg, Switzerland; julie.calonne@unifr.ch; 3 Centre Hospitalier Universitaire, 63000 Clermont-Ferrand, France; emilie.vazeille@uca.fr

**Keywords:** immobilization, recovery, mitophagy, microtubules, physical inactivity, skeletal muscle

## Abstract

Mitochondria alterations are a classical feature of muscle immobilization, and autophagy is required for the elimination of deficient mitochondria (mitophagy) and the maintenance of muscle mass. We focused on the regulation of mitochondrial quality control during immobilization and remobilization in rat gastrocnemius (GA) and tibialis anterior (TA) muscles, which have very different atrophy and recovery kinetics. We studied mitochondrial biogenesis, dynamic, movement along microtubules, and addressing to autophagy. Our data indicated that mitochondria quality control adapted differently to immobilization and remobilization in GA and TA muscles. Data showed i) a disruption of mitochondria dynamic that occurred earlier in the immobilized TA, ii) an overriding role of mitophagy that involved Parkin-dependent and/or independent processes during immobilization in the GA and during remobilization in the TA, and iii) increased mitochondria biogenesis during remobilization in both muscles. This strongly emphasized the need to consider several muscle groups to study the mechanisms involved in muscle atrophy and their ability to recover, in order to provide broad and/or specific clues for the development of strategies to maintain muscle mass and improve the health and quality of life of patients.

## 1. Introduction

Skeletal muscle provides power and strength for locomotion and posture and is the major reservoir of body proteins that can be mobilized during catabolic situations (i.e., cardiovascular diseases, cancer cachexia, chronic obstructive pulmonary diseases (COPD), chronic kidney diseases, diabetes, stressful events, etc.) to preserve vital functions. This can lead to muscle wasting, which, if too severe and/or prolonged, has adverse metabolic consequences, i.e., reduction in the effect of treatments, increased hospitalization times, and mortality [1,2]. 

Periods of immobilization or acute inactivity are often features of catabolic conditions, are inherently associated with weakness and/or frailty, and further contribute to muscle atrophy. In addition, muscle disuse is characterized by i) a decline of mitochondrial function, ii) an imbalance between mitochondrial biogenesis and degradation by autophagy (mitophagy), and iii) a decreased mitochondria abundance and/or oxidative capacity in animals and humans [3,4,5,6,7,8,9,10,11,12]. Preserving the mitochondrial function enables preventing muscle loss during muscle disuse [4,8,9,13]. In fact, the accumulation of abnormal/defective mitochondria by default of their elimination is a major contributing factor to muscle wasting [14]. Proper elimination of the damaged organelles requires i) their segregation by fission and regulation of their motility along microtubules (MTs) and ii) their addressing to autophagy through parkin-dependent and/or independent pathways [15,16,17]. Muscle disuse is associated with altered fission processes and with defective mitochondrial addressing to autophagy [7,18,19,20,21]. However, the role of mitochondrial trafficking on MTs and, more generally, the management of mitochondria alterations during recovery in skeletal muscle pending disuse are poorly documented. 

We previously used a cast immobilization rat model where the leg was fixed in plantar flexion. In that model, the gastrocnemius (GA) muscle mass decreased during immobilization and stabilized immediately after cast removal. Meanwhile, the tibialis anterior (TA) muscle moderately atrophied during immobilization, but further atrophied after cast removal. We also reported the thickening of the extracellular matrix (ECM) and activation of the mitochondria-associated apoptotic pathway in both TA and GA during immobilization and/or remobilization, suggesting a deterioration of mitochondria function [22,23]. The thickening of the ECM may reflect mechanical tension and skeletal muscle adaptations to muscle immobilization. The chaperone-assisted selective autophagy (CASA) is a tension-induced autophagy pathway that plays a critical role in mechanically strained cells and tissues [24]. In the CASA pathway, BAG3 (i.e., BCL2-associated athanogene 3) is a co-chaperone involved in the transcription of YAP1 (i.e., yes-associated protein 1), which is activated in situations of ECM stiffening or cell stretching [25] or in response to mechanical tension in vivo [24]. Regulation of YAP1 expression by mechanical tension may protect against skeletal muscle atrophy caused by denervation [25]. These markers of mechanical tension/loading are also involved in the regulation of mitochondria quality control (MQC): i) YAP1 regulates mitochondria fission during myoblast differentiation [26] and Bnip3 (i.e., BCL2/adenovirus E1B 19 kDa protein-interacting protein 3-like)-related mitophagy in microglial cells [27]. ii) BAG3 is critical for the maintenance of mitochondrial homeostasis under stress conditions in cardiomyocytes [28].

In the current study, our objective was to determine how mitochondria quality control was regulated during immobilization and remobilization in both GA and TA muscles that do not exhibit similar kinetics of atrophy and recovery. The aim was to decipher in a single study the mechanisms involved in mitochondria biogenesis, dynamic, movement along MTs, and addressing to autophagy in these two muscle groups. Addressing these questions is crucial for a better understanding of the mechanisms underlying muscle loss and recovery. 

## 2. Results 

### 2.1. Mitochondria Biogenesis Adaptations during Muscle Immobilization and Remobilization Could Not Explain Changes in Mitochondria Abundance

Table 1 shows that GA muscle mass decreased during immobilization (−24% vs. Con, *p* < 0.05) without a change in muscle fiber cross-section area (CSA) (Con: 2923 +/− 173 vs. Imm: 2768 +/− 208 µm²). During remobilization, however, GA muscle mass stabilized, while fiber CSA decreased (−19% vs. Con, *p* < 0.05). The TA muscle mass decreased during immobilization by 18% (vs. Con, *p* < 0.05). and further diminished during remobilization (−35% vs. Con and −18% vs. Imm, *p* < 0.05). We previously reported that this was associated with a decrease of TA muscle fiber CSA [22,23,29]. Mitochondria homeostasis is often deregulated during muscle disuse [3]. 

**Figure 1:**
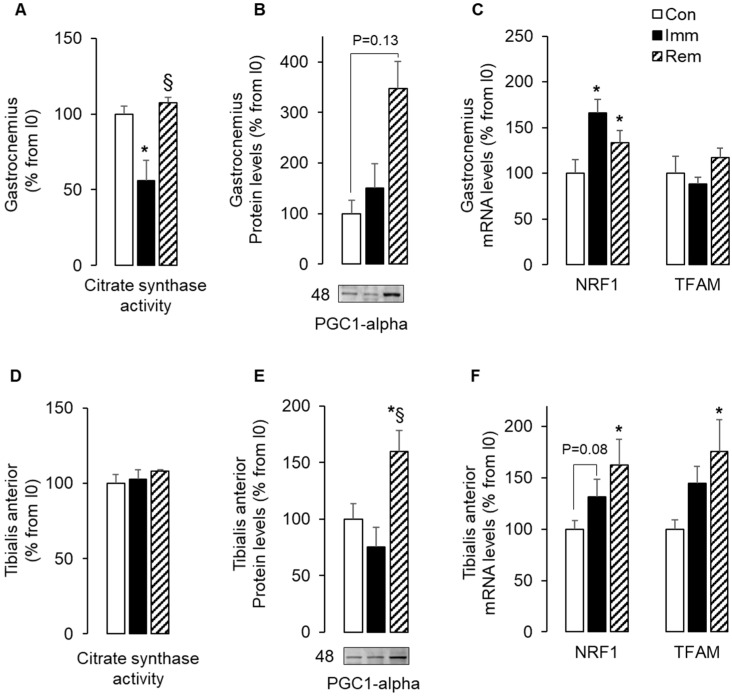
The expression of mitochondria biogenesis markers increased during remobilization. Citrate synthase activity was measured in the gastrocnemius (GA) (**A**) and the tibialis anterior (TA) (**D**), as described in Section 4. Protein levels for PGC-1α were assessed by Western blots in the GA (**B**) and the TA (**E**), quantified and normalized using Ponceau red staining for uneven loading. Representative Western blots are shown below each graph, and molecular weights are given in kDa. mRNA levels for NRF1 and TFAM were measured in the GA (**C**) and the TA (**F**) by RT-qPCR. Data were normalized using 18S rRNA. Protein and mRNA levels were expressed as % from the Con group. Statistical differences were assessed by ANOVA, as described in Materials and Methods. * *p* < 0.05 vs. Con, ^§^ *p* < 0.05 vs. Imm; Con, non-immobilized rats; Imm, immobilized; Rem, remobilized.

In accordance, Figure 1A shows that citrate synthase activity was reduced in immobilized GA (−45% vs. Con, *p* < 0.05), suggesting a decrease in mitochondria content. However, this could not be explained by changes in protein or mRNA levels for markers of mitochondria biogenesis (i.e., PGC1-α, NRF1, and TFAM). Indeed, Figure 1B,C show that PGC1-α protein and TFAM mRNA levels did not change during immobilization, whereas NRF1 mRNA levels increased (+65% vs. Con, *p* < 0.05). After 1 week of GA remobilization, citrate synthase activity returned to basal values (Figure 1A), and this was associated with elevated levels of PGC1-α protein (+250% vs. Con, *p* = 0.13) and NRF1 mRNA (+33% vs. Con, *p* < 0.05). 

The TA did not display the same changes. Figure 1D shows that citrate synthase activity did not change during TA immobilization or remobilization, suggesting that TA mitochondria abundance remained stable. Figure 1E shows that PGC1-α protein levels increased in remobilized TA muscles (+60% and 110% vs. Con and Imm, respectively, *p* < 0.05). Similarly, NRF1 and TFAM mRNA levels increased, respectively, by 63% and 76% compared to Con in the remobilized TA (Figure 1F). These data suggested that mitochondrial abundance decreased in the GA or remained stable in the TA without any reduction in mitochondrial biogenesis during immobilization or even an increase during remobilization. All these observations suggested a predominant role of mitophagy during GA immobilization and TA remobilization.

### 2.2. Mitochondria Fusion and Fission Were Imbalanced in GA and TA Muscles during Immobilization and Remobilization

**Figure 2:**
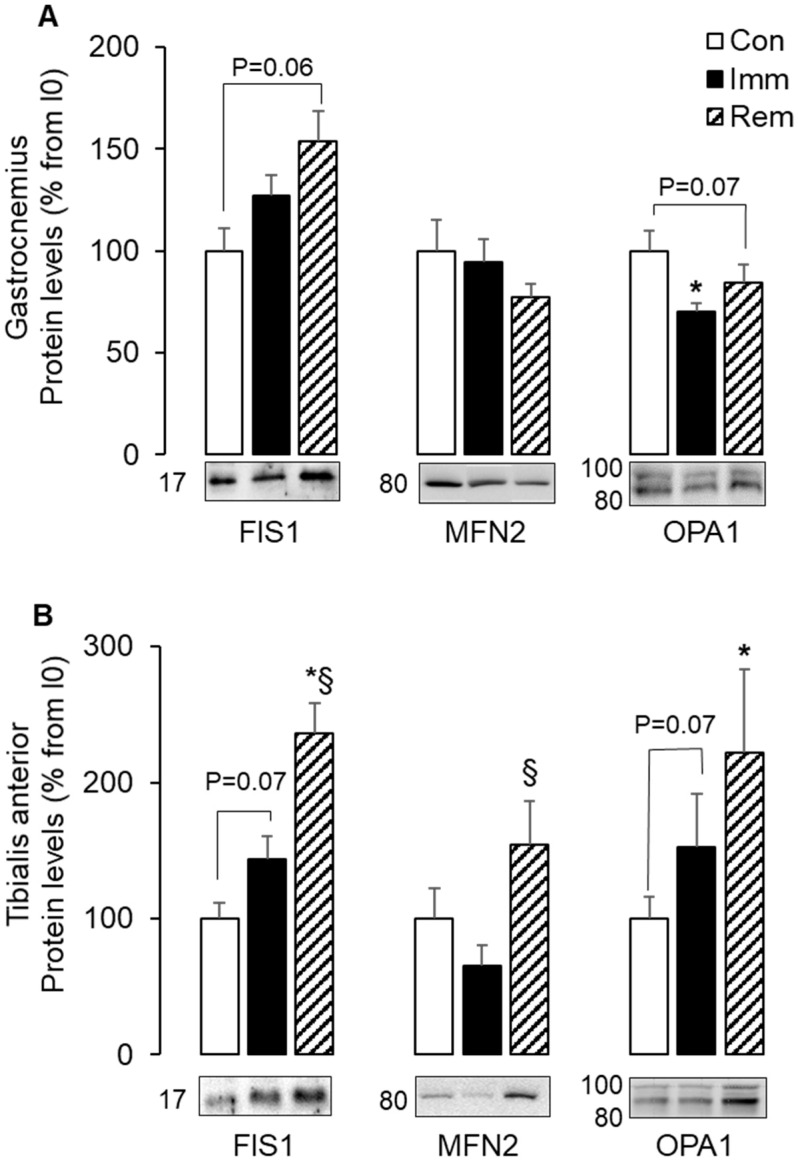
A concomitant increase of mitochondria fusion and fission markers during TA remobilization. Protein levels for FIS1, MFN2, and OPA1 were assessed by Western blots in the GA (**A**) and the TA (**B**), quantified and normalized using Ponceau red staining for uneven loading. Several isoforms of OPA1 tune mitochondrial adaptations. Thus, the 2 isoforms detected here were quantified together. Representative Western blots are shown below each graph, and molecular weights are given in kDa. Data were expressed as % from the Con group. Statistical differences were assessed by ANOVA, as described in Materials and Methods. * *p* < 0.05 vs. Con, ^§^ *p* < 0.05 vs. Imm; Con, non-immobilized rats; Imm, immobilized; Rem, remobilized.

Mitophagy is often associated with an imbalance of mitochondria fusion and fission, which are involved in the removal of damaged mitochondria. We thus investigated the impact of immobilization and remobilization on fission (FIS1, DRP1) and fusion (OPA1 and MFN2) markers. During GA immobilization, FIS1 and MFN2 protein levels did not change, and OPA1 protein levels were reduced (−30% vs. Con, *p* < 0.05) (Figure 2A). After 1 week of GA remobilization, FIS1 protein levels tended to be elevated (+53% vs. Con, *p* = 0.06), and OPA1 protein levels remained quite low (−23% vs. Con, *p* = 0.07) (Figure 2A). During TA immobilization, both FIS1 and OPA1 protein levels tended to increase (+44% and +52% vs. Con, respectively; *p* = 0.07) in (Figure 2B). After 1 week of TA remobilization, further protein level accumulation prevailed for FIS1, MFN2, and OPA1 (+136%, +54%, and +122% vs. Con, respectively, *p* < 0.05) (Figure 2B). Protein levels of DRP1 did not change during immobilization or remobilization, regardless of the muscle.

All these data indicated an imbalance in mitochondrial dynamics in the immobilized GA and in the remobilized TA and thus suggested segregation of mitochondria for their elimination by autophagy.

### 2.3. Mitochondria Trafficking along Microtubules Was Induced in Both Muscles during Immobilization and/or Remobilization

**Figure 3:**
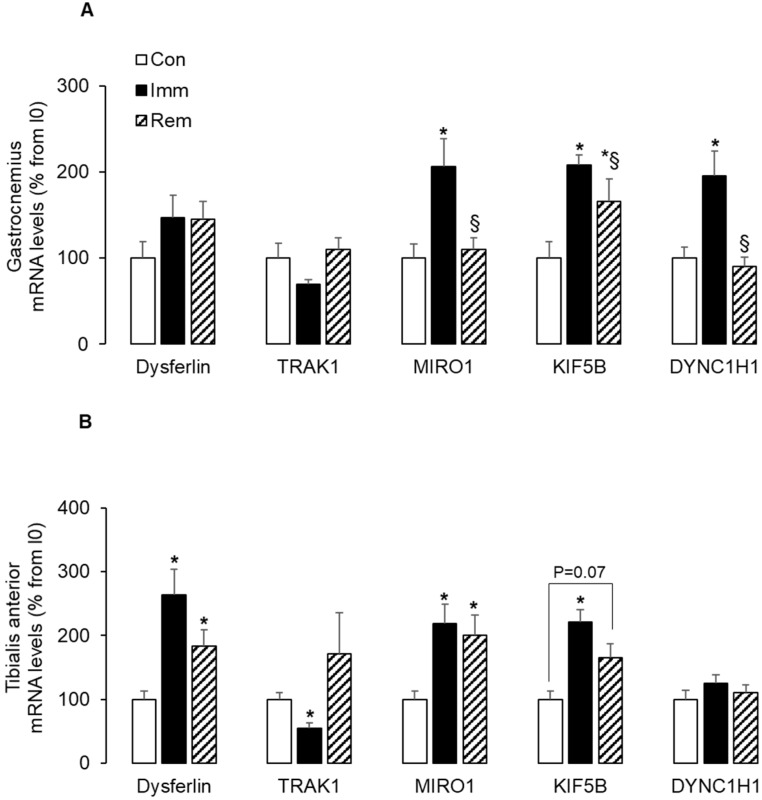
The expression of microtubules-based mitochondria trafficking markers increased during remobilization. The mRNA levels for Dysferlin, TRAK1, MIRO1, the kinesin KIF5B, and the dynein DYNC1H11 were measured in the GA (**A**) and the TA (**B**) by RTqPCR and were normalized using 18S rRNA. Data were expressed as % from the Con group. Statistical differences were assessed by ANOVA, as described in Materials and Methods. * *p* < 0.05 vs. Con, ^§^ *p* < 0.05 vs. Imm; Con, non-immobilized rats; Imm, immobilized; Rem, remobilized.

In addition to mitochondrial fission, the motility of mitochondria along microtubules (MTs) also seems to be important for mitophagy [15]. We measured mRNA levels of key markers of mitochondria movements along MTs. Dysferlin is involved in T-tubule formation and vesicle trafficking [30]. The small GTPase miro regulates mitochondrial trafficking along MTs by acting as a receptor for mitochondrial recruitment of the TRAK adaptors to drive movements mediated by the MT-based motor proteins—kinesin and dynein [31]. In the GA (Figure 3A), the mRNA levels for MIRO1, kinesin (KIF5B), and dynein (DYNC1H1) were elevated (~+100% vs. Con, *p* < 0.05) during immobilization and were either normalized (MIRO1, DYNC1H1) or reduced (KIF5B) during remobilization. A slight increase in the mRNA levels of dysferlin also prevailed during GA immobilization and remobilization. In the TA, dysferlin increased during immobilization and remobilization (+163% and +83%, respectively, vs. Con, *p* < 0.05). During TA immobilization, mRNA levels for MIRO1 and kinesin (KIF5B) increased (~+110% vs. Con, *p* < 0.05), while those for TRAK1 decreased (−45% vs. Con, *p* < 0.05). Conversely, during TA remobilization, high mRNA levels were still observed for MIRO1 (+110% vs. Con, *p* < 0.05) and KIF5B (+65% vs. Con, *p* = 0.07), while those of TRAK1 returned to control values. Dynein (DYNC1H1) mRNA levels did not change during TA immobilization or remobilization. These data suggested that mitochondria trafficking was induced along the MTs in both muscles during immobilization and/or remobilization that might enable proper addressing of mitophagy.

### 2.4. Parkin-Dependent and -Independent Mitophagy Was Activated in Muscles during Immobilization or Remobilization 

**Figure 4:**
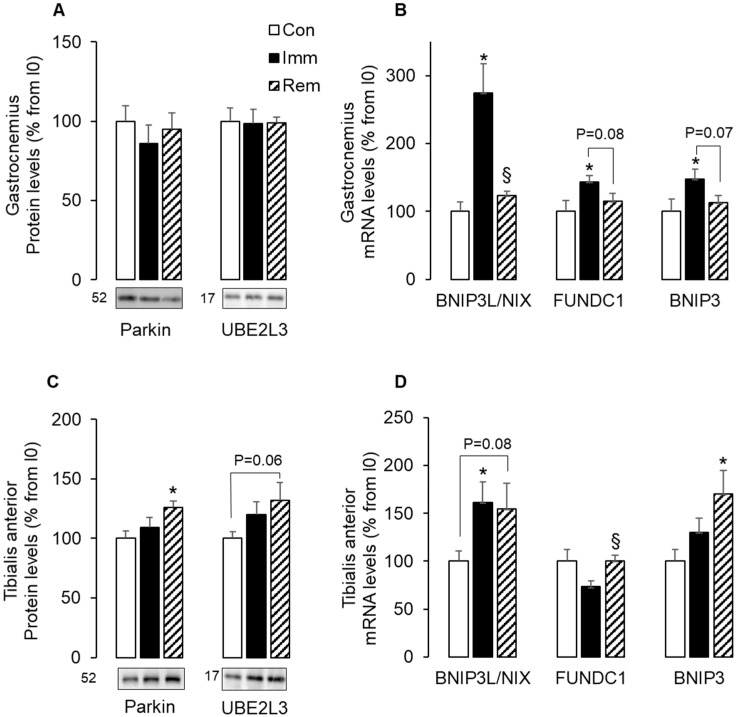
Parkin-dependent and independent mitophagy addressing pathways increased in the TA during remobilization. Protein levels for Parkin and UBE2L3 were assessed in the GA (**A**) and the TA (**C**) by Western blots, quantified and normalized using Ponceau red staining for uneven loading. Representative Western blots are shown below each graph, and molecular weights are given in kDa. mRNA levels for BNIP3L/NIX, FUNDC1, BNIP3 were measured in the GA (**B**) and the TA (**D**) by RTqPCR and were normalized using 18S rRNA. Data were expressed as % from the Con group. Statistical differences were assessed by ANOVA, as described in Materials and Methods. * *p* < 0.05 vs. Con, ^§^ *p* < 0.05 vs. Imm; Con, non-immobilized rats; Imm, immobilized; Rem, remobilized.

Cells execute mitophagy through two non-redundant mechanisms. The Parkin-dependent pathway involves the E3 ligase Parkin and the ubiquitin-conjugating enzyme—UBE2L3 [32], and the Parkin-independent pathway involves the stress-induced BNIP3, BNIP3L/NIX, and FUNDC1 molecular adaptors [33]. Immobilization of GA did not change the protein levels of Parkin and UBE2L3 (Figure 4A), but resulted in increased levels of BNIP3L/NIX (+175% vs. Con, *p* < 0.05), FUNDC1 (+43% vs. Con, *p* < 0.05), and BNIP3 (+48% vs. Con, *p* < 0.05) mRNAs (Figure 4B). After 1 week of GA remobilization, these mRNA levels returned to basal values (Figure 4B). Figure 4C shows that Parkin and UBE2L3 protein levels did not change during TA immobilization but increased after 1 week of TA remobilization (+26%, *p* < 0.05 and +33%, *p* = 0.07 vs. Con, respectively). In addition, only BNIP3L/NIX mRNA levels increased in TA muscles during immobilization (+60% vs. Con, *p* < 0.05) and remained elevated during remobilization (+54% vs. Con, *p* = 0.08) (Figure 4D). At the same time, increased mRNA levels for BNIP3 (+70% vs. Con, *p* < 0.05) and FundC1 (+37% vs. Imm, *p* < 0.05) prevailed during TA remobilization. Altogether, these data suggested that mitophagy was induced in both muscles during either immobilization or remobilization with a predominant role of the Parkin-independent mechanisms in the immobilized GA and both Parkin-dependent and independent mechanisms in the remobilized TA.

### 2.5. Autophagy Was Induced and Sustained during Immobilization and Remobilization in the TA

**Figure 5:**
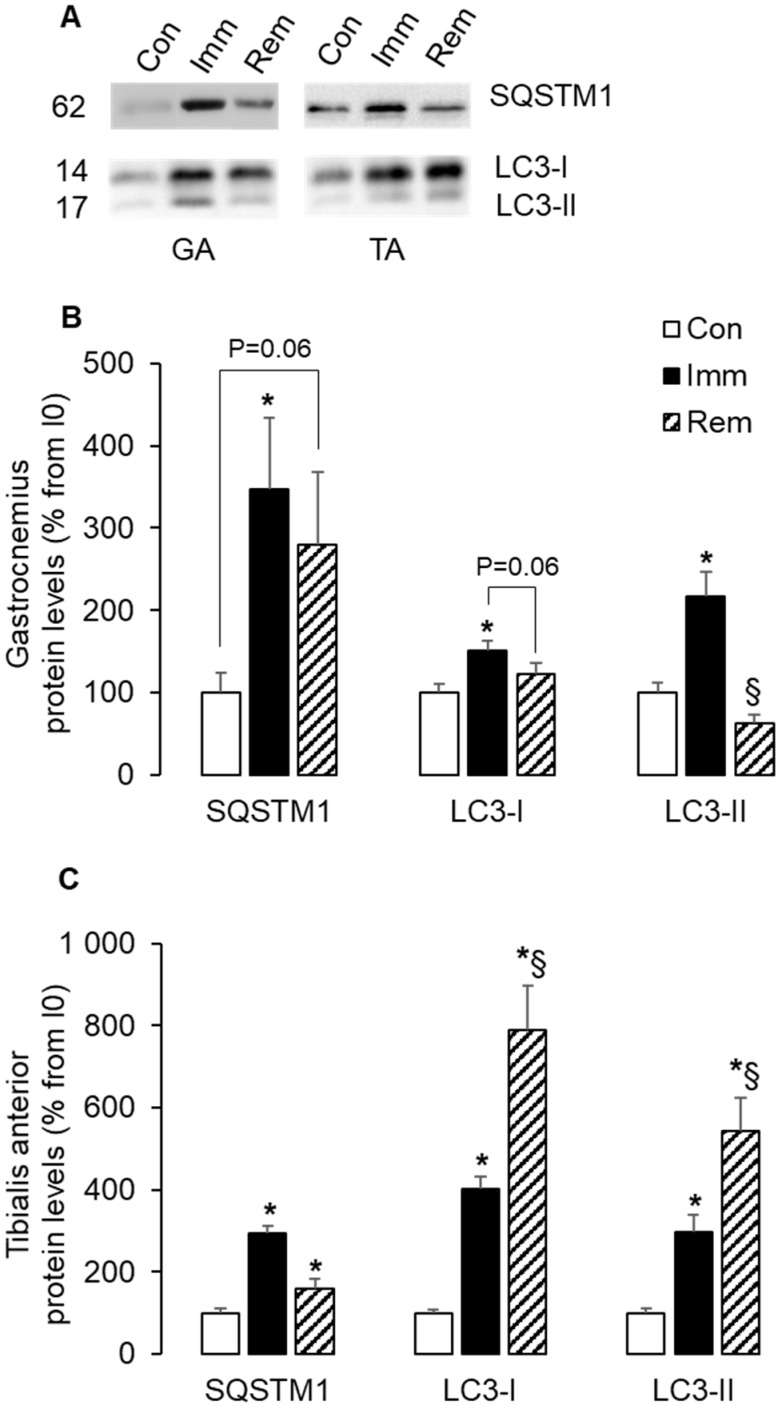
Autophagy was induced during muscle immobilization and remobilization. Protein levels for SQSTM1, LC3I, and LC3II were assessed in GA (**A**,**B**) and TA (**A**,**C**) muscles by Western blots. Two forms of LC3 were detected (LC3-I and LC3-II) and were quantified separately. Signals were quantified and normalized using Ponceau red staining for uneven loading. Representative Western blots are shown in (**A**), and molecular weights are given in kDa. Data were expressed as % from the Con group. Statistical differences were assessed by ANOVA, as described in Materials and Methods. * *p* < 0.05 vs. Con, ^§^ *p* < 0.05 vs. Imm; Con, non-immobilized rats; Imm, immobilized; Rem, remobilized.

We then investigated the regulation of autophagy in immobilized and remobilized muscles. The autophagy receptor SQSTM1 plays a central role in selective autophagy and serves as a key cargo adaptor for addressing mitochondria to mitophagy [34], and induction of autophagy requires the formation of autophagic vacuoles through lipidation of LC3 [35]. Figure 5A,B shows high protein levels for SQSTM1, LC3I, and LC3II in the immobilized GA (+247%, +50%, and +117% vs. Con, *p* < 0.05). During GA remobilization, SQSTM1 and LC3I protein levels remained slightly elevated (+180% and 23% vs. Con, *p* = 0.06). Figure 5A,C shows that SQSTM1 protein levels increased during TA immobilization (+193% vs. Con, *p* < 0.05) and remained elevated to a lower extent during remobilization (+60% vs. Con, *p* < 0.05). LC3I and LC3II protein levels rose by 2–3 fold during TA immobilization (vs. Con, *p* < 0.05) and further increased after 1 week of TA remobilization to reach a 5–7 fold induction vs. Con group (*p* < 0.05). These data showed induction of autophagy in both muscles during immobilization, which was sustained in the TA during remobilization.

### 2.6. Large and Sustained Activation of the CASA in the Immobilized and Remobilized TA 

**Figure 6:**
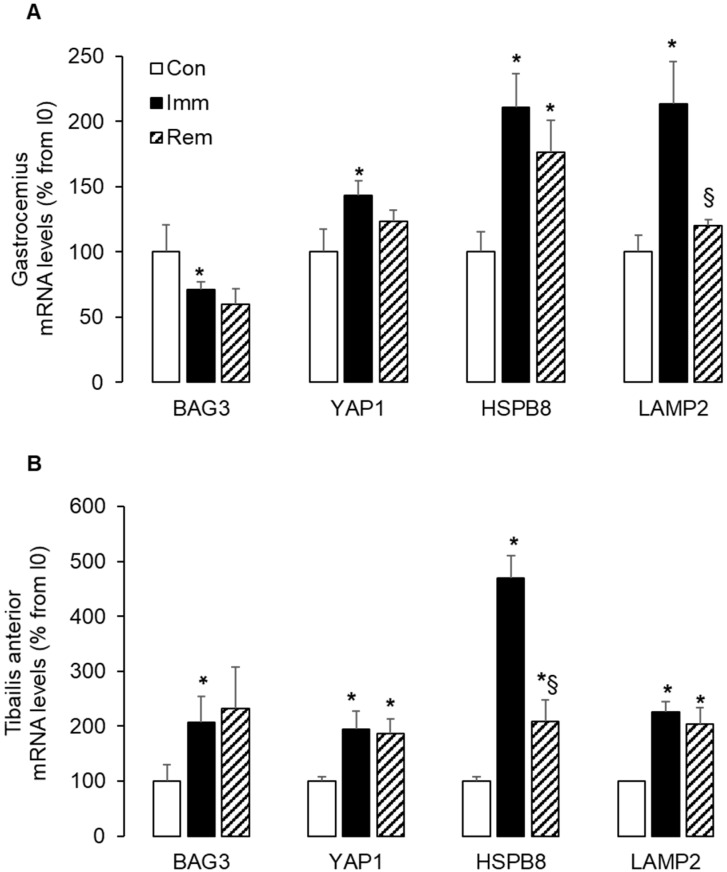
CASA (cross-section area) pathway was largely induced in immobilized and remobilized TA. The mRNA levels for BAG3, HSPB8, LAMP2, and YAP1 were measured in the GA (**A**) and the TA (**B**) by RTqPCR and were normalized using 18S rRNA. Data were expressed as % from the Con group. Statistical differences were assessed by ANOVA, as described in Materials and Methods. * *p* < 0.05 vs. Con, ^§^ *p* < 0.05 vs. Imm; Con, non-immobilized rats; Imm, immobilized; Rem, remobilized.

All these data taken together showed that the GA and TA muscles adapted differently to immobilization and remobilization. In our model, the leg had been immobilized in plantar flexion, as previously described [22,23,29]. As a result, the GA and TA muscles were, respectively, immobilized in a shortened and lengthened position. Lengthening during TA immobilization might be likened to a passive mechanical loading and might have influenced skeletal muscle adaptations to muscle immobilization. We measured the expression of markers of the chaperone-assisted selective autophagy (CASA), which is a tension-induced autophagy pathway. During GA immobilization, mRNA levels for BAG3 decreased (−29% vs. Con, *p* < 0.05), while those for YAP1, HSPB8, and LAMP2 were elevated (+43%, +110%, and +113% vs. Con, respectively, *p* < 0.05) (Figure 6A). During GA remobilization, only HSPB8 mRNA levels remained elevated (+76% vs. Con, *p* < 0.05). In the immobilized TA, the mRNA levels of BAG3, YAP1, HSPB8, and LAMP2 all raised to a greater extent and in a coordinated manner (+107%, +95%, +370%, and +126% vs. Con, respectively, *p* < 0.05) (Figure 6B). It is noteworthy that this increase sustained during remobilization but for HSPB8. Taken together, the high and persistent induction of CASA in immobilized and remobilized TA indicated that tension adaptive processes had taken place. This suggested that the TA underwent some mechanical stress during immobilization due to lengthening.

## 3. Discussion

Our main objective was to explore in a single study the regulation of mitochondrial quality control (biogenesis, mitophagy, different pathways of altered mitochondria, mitochondrial motility, autophagy) in two muscle groups (i.e., the gastrocnemius and the tibialis anterior), which have very different atrophy and recovery kinetics upon immobilization and remobilization [22,23,29]. Our data indicated that mitochondria quality control adapted differently to immobilization and remobilization in these muscles with i) a disruption of mitochondria dynamic that occurred earlier in the immobilized TA, ii) an overriding role of mitophagy during immobilization in the GA and during remobilization in the TA that involved Parkin-dependent and/or -independent processes. 

We reported here that citrate synthase activity decreased in the immobilized GA and then normalized within one week of remobilization, but remained stable in the TA. This suggested a decline in mitochondria abundance in the GA after 1 week of immobilization and no change in the TA. This was in agreement with previous reports in animal models or in humans [7,9,10,19,20], where mitochondria abundance declines during disuse in both muscles over longer durations of 2 to 4 weeks. Our study indicated that the decrease in mitochondrial abundance began as soon as 1 week of immobilization in the GA. We previously described that the proportion of type I and IIa fibers, i.e., with a high content in mitochondria, increased in response to immobilization or remobilization in the TA and the GA [22]. These changes in the composition of muscle fiber types might, therefore, have contributed to maintaining the abundance of mitochondria in the TA. However, it is unlikely that they alone have contributed to the decrease in the abundance of mitochondria in GA. Mitochondria content depends on the balance between mitochondria biogenesis and mitophagy. It is conventionally accepted that mitochondria biogenesis decreases during physical inactivity. However, the expression of biogenesis markers i) decreases over long periods of disuse (3 weeks), but is stable, reduced, or even increased in the short term [5,6,7,8,9,21,36,37,38] and ii) may be uncoordinated [5,38]. In accordance, we reported here that levels of mitochondria biogenesis markers were either stable or elevated during immobilization in both muscles. This uncoordinated regulation of markers of mitochondria biogenesis might have negatively influence the ability of mitochondrial biogenesis, as previously reported [5]. This might notably have contributed to the decline in mitochondria abundance in the immobilized GA. Conversely, our data suggested that mitochondria biogenesis was enhanced in the remobilized TA, while mitochondria abundance remained stable, suggesting very likely an overriding role of mitophagy in that muscle.

Disrupted fusion/fission dynamics is a key factor contributing to enhancing mitophagy [9,39]. In accordance, we reported here an imbalance of mitochondria dynamics in both muscles. Mitochondria fission, increased autophagy, and muscle atrophy are associated with increased FIS1 [4]. In our study, FIS1 protein levels increased only in the TA during immobilization and in both muscles during remobilization. This suggested that mitochondrial fission did not follow the same kinetics in GA and TA, with higher induction in TA being triggered during the immobilization period. However, we could not exclude increased mitochondria fission in the immobilized GA, as FIS1 may be dispensable for fission in some conditions [40]. We also showed here that mitochondrial fusion adapted differently to immobilization and remobilization in GA and TA muscles. While protein levels of MFN2 remained stable during immobilization, those of OPA1 proteins were either reduced in GA or slightly induced in TA. These discrepancies between GA and TA muscles were consistent with previous studies, where protein levels for fusion proteins after unloading or immobilization for 1 week or more were reduced in the GA [6,19] but stable in the TA [20]. In addition, we further showed that this uneven muscle response was even more pronounced during remobilization. In fact, protein levels of these fusion markers during remobilization remained low in the GA but increased in the TA beyond the values of non-immobilized control animals. This was in contrast to other reports in immobilized animals or bedridden people [20,41]. In these reports, fusion protein levels have been unchanged or low in remobilized muscles following a long-term immobilization in animals [20] or a long rehabilitation training period in humans [41]. We studied remobilization after a much shorter period of immobilization and remobilization than in these studies. This enabled us to detect muscle adaptations that could have been hidden or vanish over longer periods of immobilization and/or remobilization. Together, these data suggested that GA muscle was characterized by low fusion during immobilization and became concomitant with high fission during remobilization. Conversely, TA exhibited high fusion and fission during remobilization. This disruption in mitochondria dynamics is in favor of i) a segregation of damaged mitochondria components via fission in both muscle during remobilization with ii) a greater need for mitochondria renewal in the remobilized TA, as witnessed by the increased fusion and the coordinated induction of biogenesis markers that we reported in the TA.

Mitochondrial trafficking on MTs is also a key point for mitophagy [15]. Mitochondrial trafficking actors include i) dysferlin involved in the biogenesis and shaping of the tubule-T system, ii) the MT motor proteins dynein and kinesin for the transport of damaged and healthy mitochondria, respectively, and iii) the adaptor protein TRAK1 and the mitochondrial protein MIRO1, allowing tight coupling between MT motors and mitochondria for precise localization [15,42]. In addition, targeting TRAK1 to mitochondria can increase kinesin-mediated mitochondria transport [43]. We reported here that the expression of dysferlin increased in the immobilized TA during immobilization and remobilization, suggesting changes in the formation of the MTs. In addition, we also reported that Trak1 expression decreased during immobilization in the TA, and to a lesser extent and non-significantly in the GA. This suggested a slowing of mitochondria motility along MTs during immobilization, despite the concomitant increase in MIRO1 and in the kinesin KIF5B expression in GA and TA muscles. Both kinesin and dynein are involved i) in the late endosome and lysosome transport along MTs and ii) in recycling lysosomal membranes and membrane proteins to reconstitute a pool of functional lysosomes (i.e., lysosome tubulation) [44]. Thus, the increase in KIF5B expression that we observed in both immobilized TA and GA might also favor lysosomal motility along MTs and regulate the proper elimination of damaged mitochondria. This was consistent with the activation of autophagy that we also observed in both muscles. Finally, the concomitant increased expression of KIF5B and dynein in the immobilized GA suggested an increase in tubular lysosomes, which might evidence high consumption of lysosomes and thus a need to replenish the pool of lysosomes in that specific muscle during immobilization. 

These concomitant changes in markers of mitochondria fusion, fission, and motility in skeletal muscle during immobilization and remobilization raise the question of the distribution of mitochondria in the myofibers. Indeed, mitochondria can be localized (i) close to myonuclei and the sarcolemma (subsarcolemmal (SS) mitochondria) and (ii) between the myofibrils (intermyofibrillar (IMF) mitochondria). This distinction between SS and IMF mitochondria is not absolute, as a continuity in the network could exist: a sub-fraction could arise from the synthesis of mitochondria in another region, and the processes of fission, fusion, and organelle movement are likely to determine the location and morphology of the mitochondria. These SS and IMF mitochondria display some differences in mitochondrial respiration, enzyme activities, lipid composition, protein synthesis, and adaptation to muscle use and disuse [3]. Thus, the data reported here, indicating changes in the expression of markers involved in mitochondria dynamic (FIS1, OPA1, MFN2) and mitochondrial motility (MIRO1, TRAK1) along the microtubules (Dynein, Kinesin), supported the possibility of changes in mitochondria distribution within the myofibers that may condition mitochondrial function.

Damaged mitochondria can be addressed to autophagy through Parkin-dependent and -independent pathways [16,17,33]. Bnip3L/Nix, FundC1, and Bnip3 are mitochondrial receptors that bind directly to LC3II to bring mitochondria to the autophagosome for degradation. We reported here an overexpression of these three receptors along with increased LC3 lipidation in the immobilized GA, with no change in Parkin and UBE2L3 expression. In the TA, only Bnip3L/Nix was overexpressed with concomitantly increased lipidation of LC3 during immobilization. This strongly suggested that Parkin-independent mechanisms were at work to enhance mitophagy during immobilization in both GA and TA. These pathways were no more upregulated in the GA during remobilization, suggesting no further need for mitochondria degradation in the GA one week after cast removal. This was in accordance with a return to control mitochondria abundance (suggested by normalization of citrate synthase activity) and with the immediate arrest of GA muscle atrophy after cast removal that we previously reported [22]. Conversely, the expression of all mitochondrial receptors measured here was elevated in remobilized TA, along with elevated levels of Parkin, UBE2L3, and LC3II. This suggested that a significant need for autophagic removal of mitochondria still existed during the remobilization of the TA, via both Parkin-dependent and Parkin-independent pathways.

TA and GA muscles differ by their metabolic and contractile properties but also by their anatomical function and position. We previously reported that the proportion of fast-twitch type IIb fibers decreased in both TA and GA muscles during immobilization, whereas the proportion of slow-twitch type I and IIa fibers increased, respectively, in the GA and the TA. These changes in muscle fiber composition were intensified during TA remobilization [22]. This suggested a shift in metabolic and contractile properties towards a more oxidative metabolism during immobilization in both muscles. These changes in fiber type proportions might have influence muscle adaptations to immobilization and remobilization and thus might have contributed to or result from the additional muscle atrophy observed in the TA.

However, as both muscles undergo such metabolic differences during immobilization, their anatomical position may also exert an important constraint on the adaptation of skeletal muscles to disuse. The TA and GA muscles are antagonistic muscles: the TA is located in the anterior compartment and the GA in the posterior compartment. Accordingly, the TA is fixed in a lengthened position and the GA in a shortened position when immobilizing in plantar flexion, as in the present study. This essential difference most probably influences atrophy kinetics in both immobilized TA and GA muscles. While immobilization induces both a reduction of muscle mass and fiber CSA in the TA [23], the CSA of GA fibers was stable during immobilization, albeit a decrease of muscle mass. The decrease in fiber CSA in the TA during immobilization, whereas remaining stable in the GA, was consistent with a larger activation in the immobilized TA of the ubiquitin-proteasome system (UPS), which is involved in contractile protein degradation [22]. In addition, we previously reported that apoptotic processes were induced during GA immobilization [29,45]. This might suggest a loss of GA muscle fibers. However, the immobilized TA displays a significant decrease in fiber CSA with a moderate loss of muscle mass compared to the GA [23], as described in some previous studies [22,29,46,47,48]. We also previously reported that the ECM surrounding the muscle fibers thickened during immobilization and even more so during remobilization in the TA [22,23]. This could have minimized the impact of immobilization on the TA mass. 

The tension-induced degradation pathway CASA involves the chaperones—BAG3 and HSPB8—for the delivery of misfolded proteins to autophagy [24]. BAG3 also stimulates the transcription of the transcriptional regulator YAP1 [24] that senses mechanical tension and ECM stiffness [25,49]. In accordance, we reported here that CASA was activated during immobilization predominantly in the TA with a greater overexpression of BAG3 and HSPB8 in the lengthened immobilized TA compared to the shortened GA. This suggested a greater need for addressing altered proteins to autophagy in the immobilized TA. The activation of CASA prevailed also in the remobilized TA and thus might contribute to the further atrophy observed in this muscle after cast removal. We also reported that YAP1 expression increased during immobilization to a greater extent in the TA vs. GA and remained elevated during remobilization in the TA. Together, the regulation of these mechanical tension markers suggested that lengthening during TA immobilization could be likened to passive mechanical loading, resulting in specific muscle adaptation. 

In addition, in normal conditions, it seems that the TA tendinous tissue undertakes most of the lengthening during initial foot contact of walking and prevent potential damaging muscle stretch. Thus, the ECM thickening may lead to a decreased ability of the TA muscle-tendon to prevent muscle damage due to stretching upon muscle remobilization. This may have been exacerbated by the lengthening of the TA during immobilization. In addition, there is likely to be an acute shortening of the TA immediately after cast removal, which may constitute an atrophic stimulus, leading to the additional muscle loss that prevails immediately after remobilization. Overall, all muscle characteristics, be they metabolic, contractile, or anatomical, most likely collectively influence muscle adaptations in response to immobilization and remobilization. 

In conclusion, our data indicated a prominent role of mitophagy during immobilization and remobilization. However, the mechanisms involved in mitophagy were not the same, i.e., parkin-dependent and/or -independent, according to the nature of the muscle considered (typology of muscle fibers, anatomical position, and/or position in which they were immobilized). For example, mitophagy might explain the decreased abundance of mitochondria in immobilized GA but might contribute to increased mitochondrial turnover in remobilized TA when muscle atrophy had worsened. Finally, we also suggested that lengthening/stretching during immobilization of TA could be assimilated to passive mechanical loading, which influenced not only muscle atrophy but also muscle recovery after cast removal. This study strongly emphasized the need to consider several muscle groups, for example, according to their metabolic and contractile properties and/or their anatomical position, to study the mechanisms involved in muscle atrophy and their ability to recover. In the same vein, it would obviously be very interesting to validate the mechanisms described in this manuscript in females, as they may also display specific mechanisms and/or response kinetics, particularly in relation to hormonal cycles. This would provide broad and/or specific clues for the development of appropriated and personalized strategies to maintain muscle mass and improve the health and quality of life of patients.

## 4. Materials and Methods

### 4.1. Ethical Approval

All experiments were conducted with the approval of the regional ethics committee (agreement n° D633451, May 2017) in accordance with the European Directive 2010/63/EU on the protection of vertebrate animals used for experimental and scientific purposes. This study was performed with 33 Wistar rats aged 6 months when muscle growth is stabilized to avoid any possible bias that could arise with the use of younger growing animals. We chose to use male rats to rely on the relevant literature and on our previous work [22,23,29,45] to provide a homogeneous overall message. Rats were purchased from Charles River Laboratories (L’Arbresle, France), housed individually in the controlled room (22 ± 2 °C, 60 ± 5% humidity, 12-h light/dark cycle, the light period starting at 8 h), fed ad libitum, and given free access to water. 

### 4.2. Experimental Design

After 3-weeks of acclimatization, the rats were subjected to unilateral immobilization of the hind limb with an Orfit-soft plate (Gibaud, Saint Etienne, France) under a forene inhalation anesthesia. Rats were immobilized in plantar flexion for 1 week (Imm, *n* = 11), as described previously [22,23,29,45]. For remobilization studies, casts were then removed, and animals were allowed to recover for 1 week (Rem, *n* = 11) when differences in the kinetics of atrophy were greatest [22,23,29,45]. Immobilized and remobilized groups were compared to a group of non-immobilized rats (Con, *n* = 11). Because muscles may undergo ischemic processes during immobilization, we monitored animals daily to assess the possible occurrence of edema or inflammation. Whenever necessary, casts were removed and changed. At the end of the immobilization period, the cast did not induce significant blood flow impairment, as less than 2% of the rats had exhibited leg edema or irritation. Animals slightly reduced, moderately, their food intake during immobilization (−14% vs. Con, *p* < 0.05), without any change in body weight. During remobilization, food intake was rapidly normalized. 

### 4.3. Bodyweight and Sample Preparation

Bodyweight and food intake of rats were recorded each week during the acclimation period and every day from immobilization. At the end of the immobilization or recovery periods, animals were euthanized. Gastrocnemius (GA) and tibialis anterior (TA) muscles were carefully dissected, weighed, and frozen in liquid nitrogen. Skeletal muscles were then pulverized into a fine powder in liquid nitrogen and stored at −80 °C until further analyses. 

### 4.4. Histological Analyses of Muscles

Ten micrometer thick cross-sections of TA and GA were performed at −25 °C using a cryostat (HM500M Microm International, Fisher Scientific, Illkirch, France) and stained [22,23]. Observations and image acquisitions were performed using a photonic microscope in bright field mode (Olympus BX-51, Tokyo, Japan), coupled to a high-resolution cooled digital camera (Olympus DP72) and Cell-D software (Olympus Soft Imaging Solutions, Münster, Germany) [22,23]. After the image acquisition for each muscle section, the image analysis was performed using the Visilog 6.9 software (Noesis, Crolles, France). 

### 4.5. Quantitative Real-Time PCR Analysis

Total RNA from muscles (50 mg of muscle powder) was prepared using TRIzol reagent (Fisher Scientific, Illkirch, France) according to the manufacturer’s protocol. Determination of RNA concentration and integrity was performed using, respectively, a Nanodrop ND 1000 spectrophotometer and a bioanalyzer (Agilent Technologies, Les Ulis, France). One microgram of total RNA was treated with DNase I Amp grade (Fisher Scientific, Illkirch, France) to remove contaminating genomic DNA. Reverse transcription was performed using the superscript II reverse transcription kit (Fisher Scientific, Illkirch, France) using random primers and according to the manufacturer’s instructions. The IQ SYBR Green Supermix and the CFX96 Real-time PCR detection system (Bio-Rad Laboratories, Marnes-la-Coquette, France) were used to perform real-time PCR reactions according to the manufacturer’s instructions (see primer sequences used in Table S1). As most housekeeping genes show variations during immobilization or remobilization, the 18S rRNA was used as a reference gene (i.e., stable in our conditions). The comparative threshold cycle (2^−ΔΔCT^) method [50] was used to compare the relative mRNA expression between each group.

### 4.6. Protein Extraction

The soluble muscle proteins (100 mg of muscle powder) were extracted using a polytron in 10 vol. of an ice-cold buffer freshly prepared (pH 7.4) (20 mM HEPES, 1% triton ×100, 100 mM potassium chloride, 2 mM EGTA, 0.2 mM EDTA, 1 mM benzamidine, 50 mM β-glycerophosphate, 50 mM sodium fluoride, 1 mM dithiothreitol (DTT), 0.1 mM phenylmethylsulfonyl fluoride (PMSF), 0.5 mM sodium vanadate, and proteinase inhibitor cocktail (Sigma, Saint-Quentin-Fallavier, France)). Centrifugation of resulting homogenates was performed at 14,000× *g* (15 min, 4 °C), and protein content was measured using the Bio-Rad Protein Assay kit. Samples were prepared by dilution of aliquots of these supernatants in Læmmli buffer and stored at −80 °C until use.

### 4.7. Citrate Synthase Activity

Citrate synthase (CS) activities were measured at 37 °C on the above muscle homogenates (after storage at −80 °C) using a spectrophotometer, as described [51]. Enzyme activities were expressed in arbitrary units/min/µg muscle proteins.

### 4.8. Western Blot Analysis

Protein contents for the mitochondrial dynamin-like GTPase (OPA1), the mitofusin 2 (MFN2), the mitochondrial fission 1 protein (FIS1), the peroxisome proliferator-activated receptor-gamma coactivator 1-alpha (PGC1-α), the E3 ubiquitin-protein ligase PARKIN, the ubiquitin-conjugating enzyme E3 L3 (UBE2L3), the ubiquitin-binding protein p62 (SQSTM1), and the microtubule-associated proteins 1A/1B light chain 3B (LC3b) were assessed by immunoblotting. Briefly, proteins (30–50 µg) were separated by SDS-PAGE (sodium dodecyl sulfate-polyacrylamide gel electrophoresis) using 10 to 15% acrylamide gels and transferred onto a PVDF membrane (Immobilon-P^SQ^ 0.2 µm, Merck-Millipore, Fontenay sous Bois, France; Hybond P 0.45 µm, Fisher Scientific, Illkirch, France) using a tris-glycine-ethanol buffer (25 mM tris, 192 mM glycine, 10% ethanol, pH = 8.3). For membranes dedicated to the immunodetection of FIS1 and LC3b (i.e., with a Pi > 8.0), a CAPS (3-(cyclohexylamino)-1-propanesulfonic acid)-ethanol buffer (10 mM CAPS, 10% ethanol, pH = 11) was used to optimize protein transfer. Blots were blocked for 1 h at room temperature with 5% skim milk in TBS buffer with 0.1% tween-20 (TBS-T, pH = 7.8). For OPA1 and PGC1-α immunodetection, blots were blocked with 5% BSA (Interchim, Montluçon, France) in TBS-T. Blots were then washed three times in TBS-T and incubated (overnight, stirring, 4 °C) with the primary antibody diluted in TBS-T with 5% BSA for all antibodies except for anti-LC3, which was diluted in TBS-T with 5% skim milk. For PGC1-α immunodetection, blots were incubated 2 h at room temperature, according to the manufacturer’s instructions. The primary antibodies used in this study are described in Table S2. Blots were then washed in TBS-T and incubated for 1 h at room temperature with an appropriate secondary antibody (HRP-conjugated anti-rabbit (#7074) or anti-mouse (#7076) IgGs, from Cell Signaling Technologies) in 5% BSA or 5% skim milk in TBS-T, as for primary antibody. Detection was performed using the Luminata Crescendo or Forte Western HRP substrate (Merck-Millipore) after washing the blots three times in 1× TBS-T. Signals were visualized using the G: BOX ChemiXT4 (XL1) (Syngene, Fisher Scientific, Illkirch, France) and then quantified using the GeneTools software (Syngene). As all the classical “housekeeping” proteins (beta-actin, GAPDH…) have levels that are not stable in skeletal muscle, whether in immobilization or remobilization, signals were normalized against the amount of proteins in each lane (determined following Ponceau red staining (Sigma-Aldrich, Saint-Quentin Fallavier, France)) to correct for uneven loading. Representative images of the Ponceau red-stained membranes corresponding to the target proteins assessed in the manuscript are shown in Figure S1.

### 4.9. Statistical Analysis

All data were expressed in % variation from the Con group and were means ± SEM. Two rats were excluded from the protocol during the immobilization period because of edema or redness of their immobilized leg. Data were analyzed for normality using the Shapiro–Wilk test. No set of data was transformed for non-normality distribution. Data were analyzed by one-way analysis of variance for the effects of immobilization and remobilization in each muscle. Post hoc comparisons between groups were made using the Fisher’s PLSD test when significant differences were detected by ANOVA. The level of significance was set at *p* ≤ 0.05. All tests were performed by using XLSTAT (version 2012.4.01, AddinsoftTM, Paris, France).

## Figures and Tables

**Table 1:** GA and TA muscle mass.

	GA	TA
	Mass (g)	Mass (mg)
Con Imm Rem	2.316 ± 0.053 1.751 ± 0.051 * 1.708 ± 0.049 *	796 ± 20 656 ± 10 * 519 ± 20 *^§^

Con, control non-immobilized; Imm, immobilized; Rem, remobilized; GA, gastrocnemius; TA, tibialis anterior; CSA, cross-section area. All values are means ± SEM. * *p* < 0.05 vs. Con, ^§^ *p* < 0.05 vs. Imm. Statistics are described in the Material and Methods section.

## Supplementary Materials

Supplementary materials can be found at https://www.mdpi.com/1422-0067/21/10/3691/s1.
