# Supplementary material for: Mitophagy and Mitochondria Biogenesis Are Differentially Induced in Rat Skeletal Muscles during Immobilization and/or Remobilization
Source: Int J Mol Sci. 2020 May 23;21(10):3691. doi: 10.3390/ijms21103691 (PMC7279154; doi:10.3390/ijms21103691)
Supplement: Supplementary file 1 [file ijms-21-03691-s001.zip › Figure S1-Deval-IJMS-2020.pdf]

Figure S1

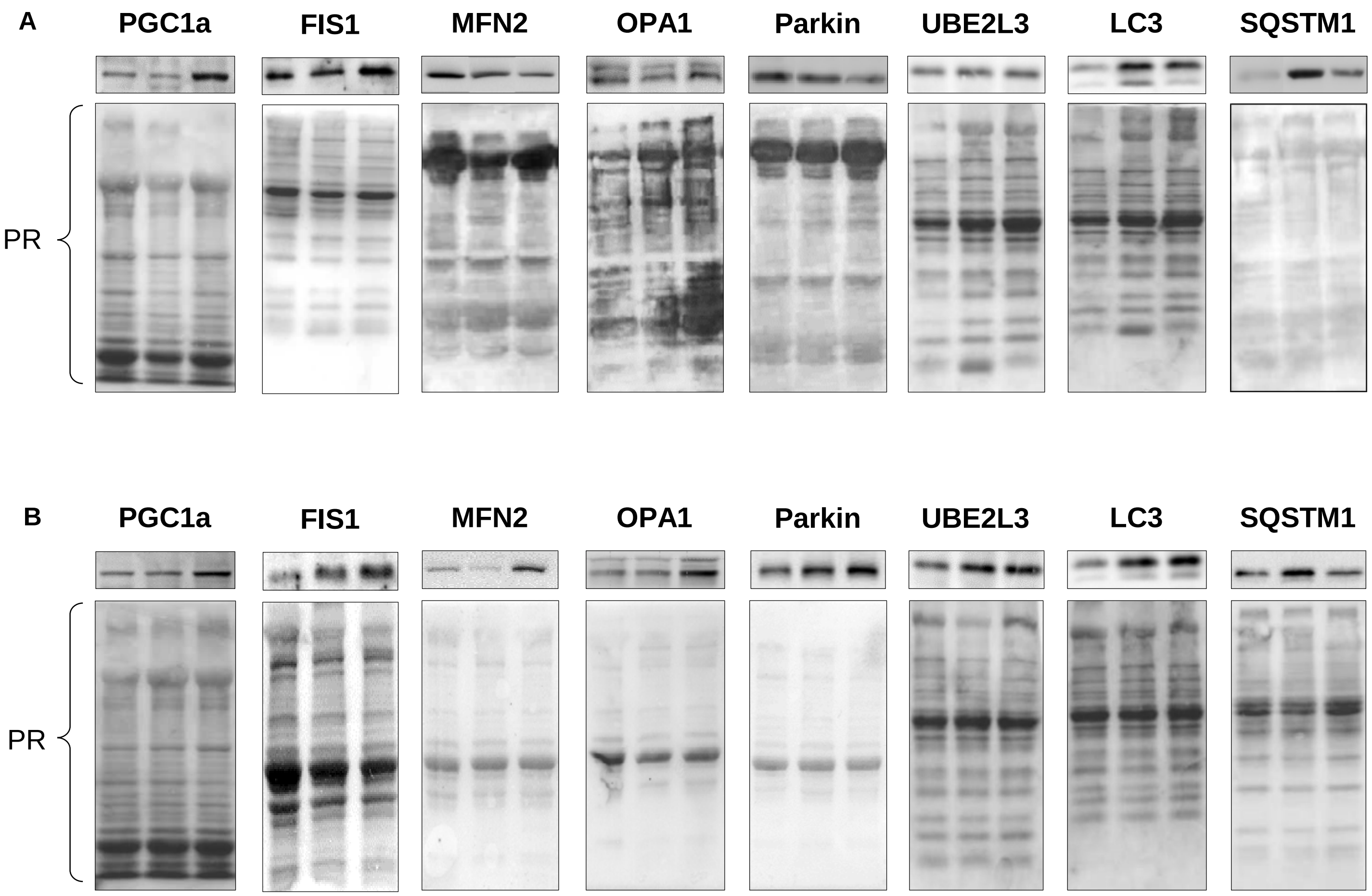

Figure S1. Ponceau red staining of membranes used for the detection of target proteins in the gastrocnemius (A) and the tibialis anterior (B) as described in material and methods section. Protein levels for each target proteins (upper panels in A and B) were assessed by Western blots in both muscles (see figures within the manuscript) and normalized using Ponceau red staining for uneven loading. Representative images for Ponceau Red (PR) staining are shown in the lower panels of A and B
