# Supplementary material for: Mitophagy and Mitochondria Biogenesis Are Differentially Induced in Rat Skeletal Muscles during Immobilization and/or Remobilization
Source: Int J Mol Sci. 2020 May 23;21(10):3691. doi: 10.3390/ijms21103691 (PMC7279154; doi:10.3390/ijms21103691)
Supplement: Supplementary file 1 [file ijms-21-03691-s001.zip › Table S1,S2.pdf]

Table S1. Primer design for the PCR assays.

Gene	Accession Number	Sequence 5′ to 3′
BAG3	NM_0010111936	***F:*** ACCGGTTCACTGCCCATC<br>***R:*** GTTTTCGGGTTGGGTGACAG
BNIP3	NM_053420	***F:*** GGACGAAGCAGCTCCAAGAG<br>***R:*** CACCAAAGCTGTGGGTGTCT
BNIP3L	NM_080888	***F:*** CAGCATGAGTCGGGACAGAG<br>***R:*** CCTGCTGGTGTGCATCTCA
DYNC1H1	NM_019226	***F:*** TCAGCAAATTTGGGCAGATG<br>***R:*** GTGTCAACGGAGTGCTGCTC
FUNDC1	NM_001025027	***F:*** GGCTGGTGTGCAGGATTTTT<br>***R:*** *CCACTGTGACTGGCAACCTG*
DYSF	NM_001107869	***F:*** ACCAGACTCGGCTACCTGGA<br>***R:*** CGGGCATTCAATATCGTCCT
HSPB8	NM_053612	***F:*** CAGCTTCCTGCAGAAGTGGA<br>***R:*** GGGTGAGTAAGGAGGGACCTG
KIF5B	NM_057202	***F:*** CCACCAGAAGAAACGAGCAG<br>***R:*** CCCTCCGGTTGCTTTACATC
LAMP2	NM_017068	***F:*** GCCTTTATGCAGAATGGGAGA<br>***R:*** TGTCACCTTGTCAGGCACTGT
MIRO1	NM_001107026	***F:*** ACAAATGAAAGCGGCTGGAT<br>***R:*** GCCCAGATACTCCAGACAACG
NRF1	NM_001100708	***F:*** GGGGCAACAGTAGCCACATT<br>***R:*** CACCTCTCCATCAGCCACAG
TFAM	NM_031326	***F:*** AATGTGGGGCGTGCTAAGAA<br>***R:*** GCTGACAGGCGAGGGTATG
TRAK1	NM_001134565	***F:*** AACCTGCGGAACAAGACGAT<br>***R:*** CGCATGGTTCCCTCAATTTC
YAP1	NM_001034002	***F:*** CATGCTCTCCCAACTGAACG<br>***R:*** TCCCATCCATCAGGAAGAGG
18S	NM_003278	***F:*** AATCAGTTATGGTTCCTTTGTCG<br>***R:*** GGTCTAGAATTACCACAGTTATCCAA

F forward, ***R*** reverse; BAG3, Bcl2-associated athanogene 3; BNIP3, BCL2-interacting protein 3; BNIP3L, BCL2-interacting protein 3-like; DYSF, DYNC1H1, Cytoplasmic dynein 1 heavy chain 1; Dysferlin; FUNDC1, FUN14 domain-containing protein 1; HSPB8, heat shock protein family B (small) member 8; KIF5B, Kinesin-1 heavy chain; LAMP2, lysosomal-associated membrane protein 2; MIRO1, Mitochondrial Rho GTPase 1; NRF1, Nuclear respiratory factor 1; TFAM, Mitochondrial transcription factor A; TRAK1, Trafficking kinesin-binding protein 1; YAP1, yes-associated protein 1.

Table S2. Primary antibodies used for Western blots.

Antibody	Dilutions	Source
Goat anti-PGC1A	1:500	Abnova, #PAB7302
Rabbit anti-MFN2	1:2000	Abcam, #Ab124773
Mouse anti-OPA1	1:1000	BD Biosciences, #612606
Rabbit anti-FIS1	1:1000	Abcam, #Ab71498
Mouse anti-PARKIN	1:10000	Sigma-Aldrich, #P6248
Anti-SQSTM1	1:5000	Interchim, #8878-M01
Rabbit anti-LC3	1:1000	Sigma-Aldrich, #L7543
Rabbit Anti-UBE2L3	1:1000	Sigma-Aldrich, #SAB2501081

PGC1A, Peroxisome proliferator-activated receptor gamma coactivator 1-alpha; MFN2, mitofusin 2; OPA1, mitochondrial Dynamin Like GTPase; FIS1, mitochondrial fission 1 protein; PARKIN, E3

ubiquitin-protein ligase; SQSTM1, ubiquitin-binding protein p62; LC3, Microtubule-associated proteins 1A/1B light chain 3B; UBE2L3, ubiquitin-conjugating enzyme E3 L3.
